# Trade Cooperation, Environmental Protection, and Sustainability: The Belt and Road Initiative Perspective

**DOI:** 10.1002/gch2.70129

**Published:** 2026-07-16

**Authors:** Yu‐Fei Zhang, Hao Wang, Li‐Jing Liu, Wen‐Hui Xu, Xiang‐Yan Qian

**Affiliations:** ^1^ Center For Energy and Environmental Policy Research Beijing Institute of Technology Beijing China; ^2^ School of Management Beijing Institute of Technology Beijing China; ^3^ Basic Science Center for Energy and Climate Change Beijing China; ^4^ Beijing Lab for System Engineering of Carbon Neutrality Beijing China; ^5^ College of Economics and Management Nanjing Agricultural University Nanjing China

**Keywords:** Belt and Road, carbon tax, CGE model, foreign investment, sustainable development, trade cooperation

## Abstract

Trade cooperation is crucial for socioeconomic development and environmental sustainability. Countries connected by the Belt and Road Initiative (BRI) account for 54% of the world's primary energy supply, and most countries along the Belt and Road (B&R) region are developing economies, typically facing the dilemma of economic growth and environmental protection. Based on the global multi‐regional dynamic computable general equilibrium model (C^3^IAM3.0/GEEPA), this study systematically simulates the impact of trade cooperation in the B&R region on the distribution of economic and environmental effects from a global economy‐wide perspective. Results show that trade liberalization fosters trade growth and enhances residents' welfare. However, it is also found that foreign direct investment has positive effects on economic growth in the mid‐and‐long term but also exacerbates environmental losses. Finally, the effectiveness of a customized carbon reduction policy is verified in this study, providing an opportunity to achieve the balance between trade cooperation and environmental protection, inspiring governments to develop a more desirable combination of policies, instead of treating economic growth and environmental protection as mutually exclusive.

## Graphical abstract

Trade cooperation in the Belt and Road region is shown to expand trade and enhance welfare, while redistributing environmental burdens across regions. This study further indicates that customized carbon policies can effectively mitigate these losses and decrease Welfare Emission Intensity, highlighting a more desirable policy pathway for aligning socioeconomic gains with environmental sustainability.

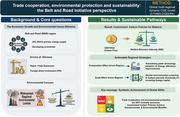

## 1 Introduction

The Belt and Road Initiative (BRI) is an international initiative that pursues openness and cooperation within the Belt and Road (B&R) region, and trade cooperation is one of the key elements. However, it involves a large number of countries and large‐scale trade, and the economic characteristics and development levels of such countries are uneven. While the impact of trade cooperation may be extensive and profound, it also comes with great uncertainty. Particularly, since the BRI was put forward, there has been a lot of attention regarding its possible environmental impact.

At present, since the global economy is closely connected, the evolution of the international trade pattern brought about by new trade cooperation affects the socio‐economic development of all countries. The global economic recession caused by the coronavirus disease (COVID‐19) further highlights, more than ever, the importance of international cooperation in keeping the global market for goods and services open [1, 2, 3, 4]. For countries in the B&R region, on the one hand, socio‐economic benefits such as economic development and welfare creation, especially for developing countries which are in urgent need of dealing with poverty (SDG 1), hunger and food insecurity (SDG 2), and access to clean and affordable energy (SDG 7) are important motivation in their participation in trade cooperation; on the other hand, both the worrying emission current situation in the B&R region and the countries’ endowed international obligations of climate action(SDG 13) magnify the necessity of paying attention to environmental protection through economic and trade cooperation. Therefore, in the face of potential gains and losses, how to better balance socio‐economic development and environmental protection has become a core issue for the synthetical realization of SDGs on a global scale.

A considerable number of studies have discussed the relationship between the economy and the environment in the context of the BRI. These can be divided into the following categories: First, determining the relationship between economic development trends/characteristics and environmental conditions, which is often accompanied by a verification of the environmental Kuznets curve hypothesis [5, 6, 7, 8]; second, accounting for embodied emissions from production and trade activities [9, 10, 11]; third, assessing the environmental risks associated with specific economic activities, like infrastructure construction [12, 13, 14]; and fourth, checking the environmental conditions of the atmosphere, land use, biodiversity, and so forth, or calculating specific environmental indicators and exploring their economic impact factors [15, 16, 17, 18]. Among the existing studies, the research focusing on trade cooperation in the B&R region is mainly divided into two parts: First, econometric analysis based on historical data, that is, using trade openness or trade volume as one of the economic development indicators, the relationship between trade and environmental indicators is analyzed. However, due to the differences in the index system, data range, model setting, and other aspects, the conclusions of the relevant studies are inconsistent. More recently, to address these inconsistencies, an emerging stream of literature has shifted focus toward “Green BRI” development, explicitly investigating how green finance, renewable energy investments, and green foreign direct investment (FDI) can decouple economic growth from carbon emissions during B&R trade expansion [19, 20]. Second, research on the embodied emissions of trade in the B&R region can reveal the direction and amount of emissions transfer. However, such research is usually based on input‐output models and focuses on describing the emissions transfer contained in the trade chain in the B&R region. Thus, these studies are unable to clearly characterize the changes in international trade and related emission footprints before and after the implementation of the BRI. They are also unable to fully present the causes of emissions changes in various regions during trade cooperation.

Apart from the research focused on the BRI, many studies in the past few decades have focused on the evaluation of the impact of trade cooperation in other regions. However, its reference significance for understanding the impact of B&R trade cooperation and designing feasible supporting programs is limited. On the one hand, the relationship between trade cooperation and environmental quality is never certain and is always affected by national policies [21], particularly the recent global emphasis on carbon pricing mechanisms and emission trading schemes [22, 23, 24], production technology [25], income level [26, 27] and even personal choice [28, 29]. On the other hand, the BRI involves a large number of countries and has a wide coverage, and the economic development levels of such countries are uneven. Since the openness of the initiative makes the process more complicated and uncertain, it is necessary to carry out a targeted evaluation that incorporates effective environmental policy simulations, such as carbon taxes.

Compared with existing studies, this study is an improvement in several aspects: first, the economic and environmental benefits and losses of B&R trade cooperation in the medium and long term are comprehensively simulated and analyzed; and second, using sustainable cooperation and development as the starting point, further discussions are had on the feasibility and possible solutions of balancing socio‐economic development and environmental protection.

This study focuses on the following core questions: How will the socio‐economic and environmental impacts of the B&R trade cooperation process be distributed? Faced with potential gains and losses, is there a feasible solution that can balance socio‐economic development and environmental protection? Focusing on the core issues above and based on a global multiregional dynamic computable general equilibrium (CGE) model, this study designs six scenarios by combining the cooperation vision of the BRI and China's development strategies for foreign trade; simulates multiple trade cooperative options in the context of the BRI; assesses its impact on socio‐economic indicators and major greenhouse gas (GHG) emissions (CO_2_, CH_4_, and N_2_O) in the medium and long term; and presents the distribution of economic and environmental gains and losses in various regions on a global scale. Furthermore, in view of potential environmental losses in different regions, carbon tax policies with different carbon price levels and carbon tax revenue recycling methods are introduced into the model to verify the existence and effectiveness of acceptable environmental policies. Finally, combined with a composite indicator (GHG emissions per unit of resident welfare) we developed, the coordination of socio‐economic development and environmental protection in B&R trade cooperation is discussed, and policy recommendations are given.

## 2 Methods

### 2.1 China's Climate Change Integrated Assessment Model/Global Energy and Environmental Policy Analysis (C^3^IAM3.0/GEEPA) Model

**FIGURE 1:**
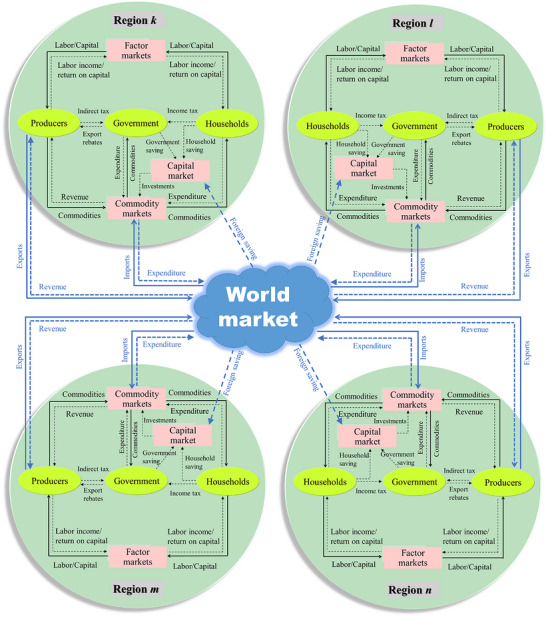
The framework of China's climate change integrated assessment model/global energy and environmental policy analysis (C^3^IAM3.0/GEEPA) [35].

The model used in this study is China's Climate Change Integrated Assessment Model/Global Energy and Environmental Policy Analysis (C^3^IAM3.0/GEEPA), which was developed by the Research for Energy and Environmental Policy of Beijing Institute of Technology. As the core economic module of C^3^IAM3.0/GEEPA is a global multiregional recursive dynamic CGE model and has been applied to research on the economic and environmental impacts of trade frictions [30] as well as international cooperation in reducing emissions [31]. GEEPA is composed of five basic modules: production, income, expenditure, investment, and foreign trade modules. The basic framework of the model is shown in Figure 1. The basic assumptions and mathematical expressions of each module are shown in Wei et al. [32]. The basic socio‐economic data of the GEEPA model comes from the Global Trade Analysis Project (GTAP) database [33]. Additionally, GEEPA covers the main GHG emissions (CO_2_, CH_4_, and N_2_O). The GHG emission data for the base year comes from the Greenhouse Gas and Air Pollution Interactions and Synergies (GAINS) database [34].

To focus on the B&R region further in this application of the GEEPA, we divide the world into 13 regions: China (China), Russia (Russia), the Association of Southeast Asian Nations (ASEAN), Central Asia (CA), South Asia (SA), Other East Asia (OEA), the Middle East (ME), Eastern Europe (EE), the United States (USA), Latin America (LAM), other European countries (OEU), Africa (Africa), and the rest of the world (ROW) (see Supporting Information S1). This study includes 20 sectors (see Supporting Information S2 for details).

In the import trade module of C^3^IAM3.0/GEEPA, the commodity in one region that is supplied domestically is composed of domestic and imported commodities following a nested constant elasticity of substitute (CES) function, shown in Equation (1).

(1) $$\left\{\begin{array}{c} Q_{i}=\alpha_{Mx,i}{\left(\beta_{M,i}M_{i}^{\rho_{Mx}}+\beta_{D,i}D_{i}^{\rho_{Mx}}\right)}^{\frac{1}{\rho_{Mx}}}\\ M_{i}={\left[\frac{\alpha_{Mx,i}^{\rho_{Mx}}\beta_{M,i}P_{i}^{Q}}{P_{i}^{M}\left(1+\tau_{i}^{M}\right)}\right]}^{\frac{1}{1-\rho_{Mx}}}Z_{i}\\ D_{i}={\left[\frac{\alpha_{Mx,i}^{\rho_{Mx}}\beta_{D,i}P_{i}^{Q}}{P_{i}^{D}}\right]}^{\frac{1}{1-\rho_{Mx}}}Z_{i} \end{array}\right.$$

where α_*Mx*,*i*_represents the shift parameter, β_*M*,*i*_ represents the share parameter of importing good *i*, β_*D*,*i*_ represents the share parameter of domestic good *i*, *M_i_* and *D_i_*, respectively, represent imports and domestic produced good *i*,ρ_*Mx*_ represents the substitution parameter between import and domestic goods, *P_i_* represents the price of good *i*, and $\tau_{i}^{M}$ represents the import tariff of good *i*.

C^3^IAM3.0/GEEPA adopts the principle of foreign savings set exogenous, and the exchange rate set endogenous. Foreign savings are the difference between the income and expenditure of foreign currency in one region. Foreign direct investment (FDI) is represented through changes in foreign savings, which increase the investment resources available in recipient economies while reducing domestic savings in the source economy. Additional investment contributes to capital formation and is incorporated into the recursive dynamic capital accumulation process. Specifically, capital stocks associated with overseas investment evolve according to a perpetual‐inventory approach, where newly created capital is added to the existing stock after accounting for depreciation. To reflect the time required for investment projects to become operational, newly formed capital does not generate returns in the year of investment. Instead, investment affects production capacity and factor returns from the subsequent period onward. Capital income generated by overseas investments is assumed to accrue to the investing country. The share of capital income attributed to foreign‐owned capital is determined by the ratio of accumulated FDI‐related capital stock to total regional capital stock. Corresponding adjustments are made to regional capital income and balance‐of‐payments accounts to ensure accounting consistency within the general equilibrium framework. A limitation of this treatment is that FDI is represented mainly as an aggregate capital flow. The model does not explicitly distinguish sector‐specific FDI inflows or capture potential technology spillovers, management improvements, and productivity effects associated with foreign investment.

### 2.2 Scenarios

According to the “Initiative on Promoting Unimpeded Trade Cooperation along the Belt and Road ” [36], the main measures for trade cooperation within the framework of the BRI are to promote the liberalization and facilitation of trade and investment. Trade opening is one of the key elements. Furthermore, the revitalization of investment is another key objective and is an important measure to promote trade exchanges. Hence, this study focuses on trade opening and FDI, and develops six scenarios to simulate various means of trade cooperation in the context of the BRI.

#### 2.2.1 Trade Opening Scenario

First, this study constructs two open trade scenarios, namely the bilateral trade agreement (BTA) scenario and the multilateral trade agreement (MTA) scenario. Since there is no specific top‐level design or cooperation plan for trade opening, this study combines China's top‐level development strategies and phased goals for foreign trade, and focuses on three aspects of scenario design: first, the point in time when China and other countries reach free trade agreements; second, the order in which China reaches free trade agreements with other countries; and third, the tax reduction pattern between different countries.

Regarding the point in time for reaching a free trade agreement, the Ministry of Commerce of China proposed phased goals for trade development based on the strategic plan of the 19th National Congress of the Communist Party of China: China will further consolidate its status as a major economic and trade country by 2020; basically and comprehensively become a powerful economic and trade country by 2035 and by 2050, respectively. Based on this, this study sets 2035 and 2050 as the time node for reaching a free trade agreement.

In terms of the sequence of regions for reaching free trade agreements, combined with China's strategic plan to “build a network of high‐standard free trade zones based on the periphery, radiating the ‘B&R’ and facing the world,” this study assumes that China will give priority to advancing free trade agreement negotiations with neighboring countries, followed by other countries along the B&R. 2020 and 2035 are regarded as the time points for China to reach the free trade agreements with neighboring countries and other B&R countries respectively. Because of complex political and economic relations as well as many challenges that may entail a long and uncertain process to reach global trade cooperation, this study will not consider the establishment of a global free trade area for the time being.

In terms of tax reduction patterns, this study will consider two situations: (1) bilateral tax reduction, that is, China and neighboring countries and other B&R countries will reach BTAs in 2020 and 2035, respectively, and China and other countries will mutually exempt tariffs; and (2) multilateral tax reduction, that is, China and its neighboring countries and other B&R countries will reach an MTA in 2020 and 2035, respectively, to establish a zero‐tariff free trade zone in their corresponding regions. Due to the uncertainty of tariff reduction, this study assumes the greatest impact, which is tariff exemption. The corresponding simulation results can help decision‐makers clarify the upper limit of economic and environmental impacts.

Finally, in the BTA scenario, China and its neighboring countries will be exempted from each other's tariffs in 2020, and China and other countries along the B&R will be exempted from each other's tariffs in 2035; in the MTA scenario, China and its neighboring countries will establish a free trade zone in 2020, and China, neighboring countries, and other countries along the B&R will establish a free trade zone in 2035. Considering that 2050 is the target year for China to conclusively become an economic trade power, the simulation period for this study is from 2020 to 2050.

#### 2.2.2 Trade‐Investment Scenario

In consideration of the close relationship between investment and trade within the framework of the BRI, this study introduces FDI based on BTA and MTA scenarios, and further constructs trade‐investment scenarios where trade and investment coexist, which can simulate the process of China's direct investment in other B&R countries when tariff exemption occurs. At the same time, both small‐ and large‐scale investments will be considered.

This study establishes four trade‐investment scenarios: for the BTA‐FDI‐1% and MTA‐FDI‐1% scenarios based on the original BTA and MTA scenarios, the foreign savings of all the B&R routes except China will be increased by 1% of the total amount of new investment that year; at the same time, the total increase in savings of all FDI inflow countries will correspondingly be subtracted from China's foreign savings. In this way, the investment inflows from China and other participants can be simulated. In the BTA‐FDI‐5% and MTA‐FDI‐5% scenarios, the scale of investment rises to 5% of the total new investment volume in each region that year, and other settings remain unchanged.

In summary, this study constructs a total of six trade cooperation scenarios (see Table 1). As a benchmark reference, an additional business as usual (BAU) scenario that always maintains the original tariff level and does not introduce any other shocks is also set, so that the numerical results of the trade cooperation scenarios can be compared with, and the economic and environmental impacts of trade cooperation in the B&R region can be determined.

**TABLE 1:** Scenario design.

	Trade‐related setting			FDI‐related setting
Scenario	Launch year	Country scope	Tax reduction pattern	
BAU	NA	NA	NA	NA
BTA	2020	Neighboring countries^a^	China reaches bilateral cooperation agreements with corresponding countries to exempt tariffs from each other	No FDI
	2035	Other countries along the B&R ^b^		
MTA	2020	Neighboring countries	A multilateral agreement is reached between the member states of the free trade area to establish a zero‐tariff free trade area	No FDI
	2035	Other countries along the B&R		
BTA‐FDI‐1%	Same as BTA			The foreign savings of all the B&R regions except China increases by 1% of the new investment volume in the year, and China's foreign savings is subtracted by the total increase in foreign savings in each region.
MTA‐FDI‐1%	Same as MTA			
BTA‐FDI‐5%	Same as BTA			The foreign savings of all the B&R regions except China increases by 5% of the new investment volume in the year, and China's foreign savings is subtracted by the total increase in foreign savings in each region.
MTA‐FDI‐5%	Same as MTA			

*Note*: B&R, Belt & Road; BAU, business as usual; BTA, bilateral trade agreement; FDI, foreign direct investment; MTA, multilateral trade agreement

^a^ Neighboring countries include Russia (Russia), South Asia (SA), the Association of Southeast Asian Nations (ASEAN), Central Asia (CA), and other East Asian countries (OEA).

^b^ Other BRI countries include the Middle East (ME) and Eastern European (EE).

## 3 Results

### 3.1 Trade Expansion, Economic Growth, and Welfare Creation

**FIGURE 2:**
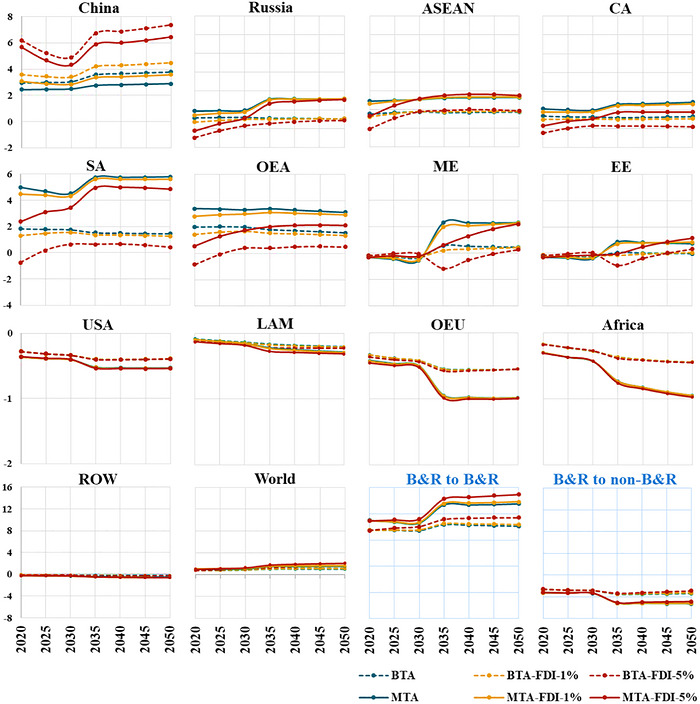
Percentage change in global and regional trade volume (%). *Note*: ASEAN, the Association of Southeast Asian Nations; CA, Central Asia; SA, South Asia; OEA, Other East Asia; ME, the Middle East; EE, Eastern Europe; USA, the United States; LAM, Latin America; OEU, other European countries; ROW, the rest of the world; B&R, Belt and Road.

Trade openness in the B&R region promotes the expansion of overall trade scale among participating regions and globally. However, it also triggers a trade diversion effect. It is also found that multilateral trade opening has a stronger role in promoting trade creation and diversion, and large‐scale FDI inflows are not conducive to exports. Compared with BAU, each participating region will experience an increase in export volume, with global trade volume rising by approximately 0.80%–1.02% (BTA) and 1.02%–1.53% (MTA), respectively. The trade diversion effect is manifested in the trade transfer from the trade between B&R and non‐B&R regions to that within the B&R regions. Under the BTA and MTA scenarios, the B&R regions will experience a significant increase in trade exchanges, with a growth rate of 5.48%–6.35% and 6.61%–9.56%, respectively. However, there will be a decrease in trade volume between the B&R and non‐B&R regions, with a decline of 2.64%–3.48% and 3.24%–5.27%, respectively. Multilateral trade opening promotes greater trade prosperity and transfer compared to bilateral trade opening (see Figure 2). Additionally, FDI may be detrimental to the exports of the inflowing country. This is because, on the one hand, FDI will inhibit exports by increasing the exchange rate of the inflowing country. On the other hand, FDI will increase the domestic final demand of the inflowing country, thereby having a substitution effect on exports. When introducing 1% FDI (BTA‐FDI‐1% and MTA‐FDI‐1%), the export volume of each inflowing country shows a slight downward trend compared to the scenario without FDI. With the introduction of 5% FDI (BTA‐FDI‐5% and MTA‐FDI‐5%), FDI nearly offsets the trade creation effect resulting from bilateral trade opening. This leads to a situation where the export volume of certain countries in the BTA‐FDI scenario is lower than the BAU level. Under the MTA‐FDI scenario, however, the export volume of each inflowing country will maintain a positive growth rate. FDI will promote the increase in China's export volume, which will enable the overall export volume of the B&R region to remain higher than BAU.

**FIGURE 3:**
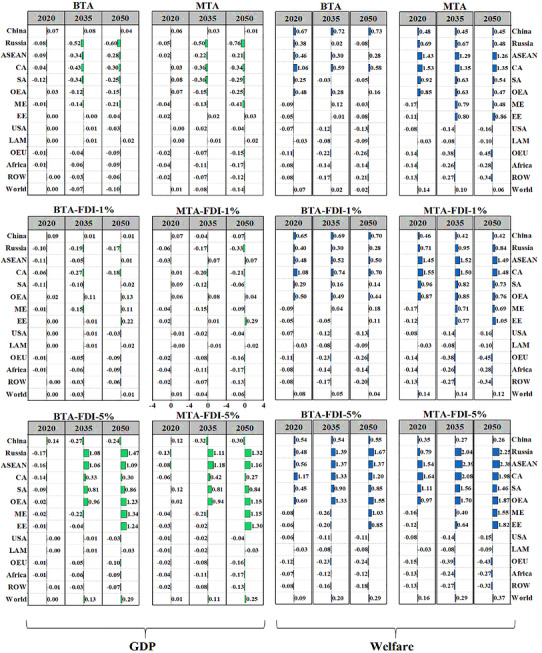
Percentage change in global and regional gross domestic product (GDP) growth and welfare (%). *Note*: BTA, bilateral trade agreement; MTA, multilateral trade agreement; FDI, foreign direct investment; ASEAN, the Association of Southeast Asian Nations; CA, Central Asia; SA, South Asia; OEA, Other East Asia; ME, the Middle East; EE, Eastern Europe; USA, the United States; LAM, Latin America; OEU, other European countries; ROW, the rest of the world.

Trade opening and economic trade cooperation in the B&R region will improve the overall welfare level of residents in both participating regions and the whole B&R region, as seen in Figure 3. Compared with the BAU scenario, the residents’ welfare in the B&R region (excluding China) shows significant improvement under different scenarios. Under the BTA scenario, the welfare increases by 0.16%–0.73% in 2050, which further rises to 0.11%–0.70% with the introduction of 1% FDI (BTA‐FDI‐1% scenario) and 0.55%–1.67% with the introduction of 5% FDI (BTA‐FDI‐5% scenario). Similarly, Under the MTA scenario, the residents' welfare in the B&R region increases by 0.45%–1.35%, which further rises to 0.42%–1.49% with the introduction of 1% FDI (MTA‐FDI‐1% scenario) and 0.26%–2.38% with the introduction of 5% FDI (MTA‐FDI‐5% scenario). Therefore, multilateral trade cooperation is more conducive to the improvement of residents’ welfare in the B&R countries than bilateral trade cooperation, and the introduction of FDI is conducive to further improving the welfare of residents.

As shown in Figure 3, trade opening in the B&R region has a very weak impact on economic growth, while the coexistence of trade and FDI in trade cooperation is conducive to economic growth. Compared with BAU, the change in gross domestic product (GDP) of each participating region is no more than 0.1% in trade opening scenarios (BTA and MTA). The reason is that the exemption of tariffs leads to a sharp drop in government tariff revenue, thereby reducing societal demand to a certain extent. The inflow of FDI will help increase new social investment and make up for the negative impact of the sharp drop in government tariff revenue. As can be seen from Figure 3, with a 5% increase in China's investment in each region, the GDP of the B&R regions is expected to grow by 0.30%–1.47% (BTA‐FDI‐5%) and 0.27%–1.32% (MTA‐FDI‐5%) by 2050.

### 3.2 Weak Impact on Global Greenhouse Gas (GHG) Emissions

There is no significant increase or decrease in total global GHG emissions, indicating that trade cooperation in the B&R region will not bring significant pressure on global action to combat climate change. Under the open trade scenarios (BTA and MTA) and the scenarios of introducing low‐level FDI (BTA‐FDI‐1% and MTA‐FDI‐1%), the total GHG emissions will not change by more than 0.1% in the short and long term. Larger‐scale FDI has a more significant role in stimulating production and consumption—resulting in more emissions. However, the increase in total global GHG emissions in the BTA‐FDI‐5% and MTA‐FDI‐5% scenarios remains below 0.15%.

### 3.3 Regional Emission Performance and Governance Strategy

**FIGURE 4:**
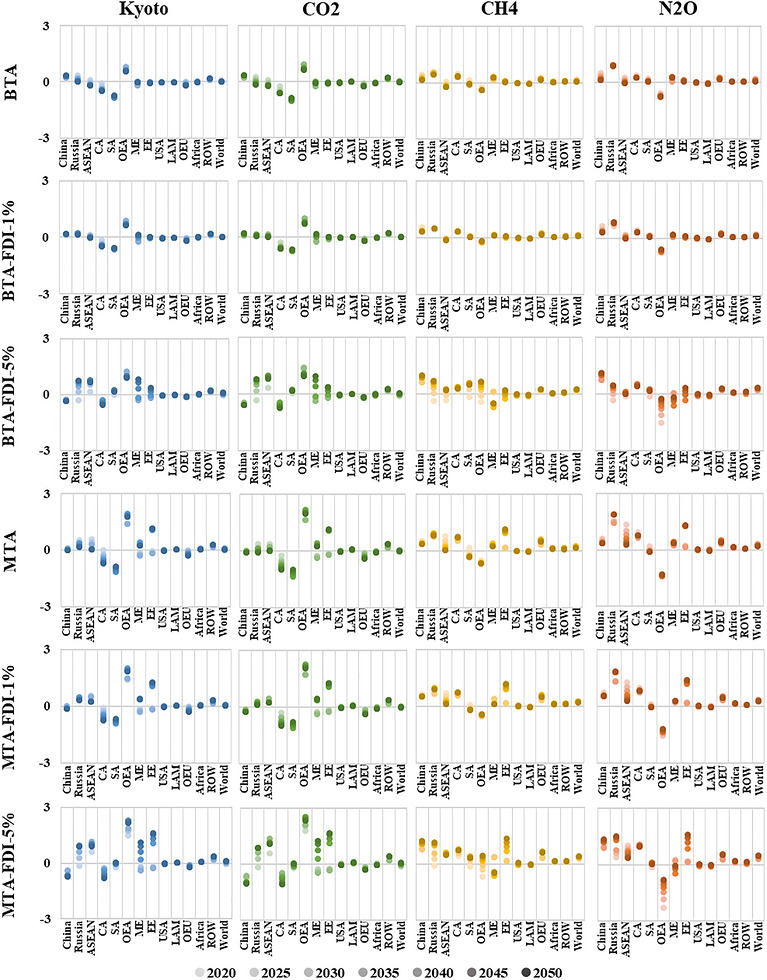
Percentage change in global and regional greenhouse gas (GHG) emissions (%). *Note*: MTA, multilateral trade agreement; FDI, foreign direct investment; BTA, bilateral trade agreement; ASEAN, the Association of Southeast Asian Nations; CA, Central Asia; SA, South Asia; OEA, Other East Asia; ME, the Middle East; EE, Eastern Europe; USA, the United States; LAM, Latin America; OEU, other European countries; ROW, the rest of the world.

Trade liberalization will trigger emissions transfer, which will change the emission pattern of the B&R region to a certain extent, causing individual regions to bear environmental losses. As shown in Figure 4, under bilateral trade opening, GHG emissions in other East Asia regions will increase by 0.55%–0.80% annually compared to BAU. South Asia will experience a significant reduction in GHG emissions by 0.68%–0.84% annually. Under the multilateral trade opening scenario, other East Asia and Eastern Europe will see a substantial increase in GHG emissions (over 1% annually compared to BAU). South Asia will have a significant reduction in GHG emissions by 0.87%–1.15% annually, while Central Asia will experience a notable annual reduction of 0.50%–0.71% in the medium to long term (2035–2050). The GHG emissions of other B&R regions will show a slight increase or decrease to varying degrees, while the GHG emissions of non‐B&R regions will remain relatively unchanged.

Large‐scale FDI will result in increased emissions from inflowing countries, causing more subregions to experience environmental losses. As shown in Figure 4, the introduction of 1% FDI (BTA‐FDI‐1% and MTA‐FDI‐1% scenarios) is projected to result in a relatively minor increase in emissions, with no significant changes observed in the emission pattern. Other East Asia and Eastern Europe continue to be the primary regions experiencing environmental losses, while South Asia and Central Asia remain the main regions witnessing reductions in emissions. 5% FDI (BTA‐FDI‐5% and MTA‐FDI‐5% scenarios) will significantly increase the emission level of each inflow region, thereby expanding the coverage of environmental damage, and making other East Asia, Eastern Europe, Russia, ASEAN, and the Middle East all become regions of increased emissions (relative to BAU). At the same time, the long‐term emission reduction in South Asia will disappear in the trade opening scenario.

This study applies the decomposition method, referring to [30, 37], to analyze the changes in GHG emissions across different regions. The decomposition analysis separates the GHG emission changes into three factors: scale effects (changes in output size), composition effects (changes in output share), and technique effects (changes in emission intensity), see Supporting Information S3 for the decomposition results. The analysis reveals that the composition effect plays a leading role in the changes in the emission pattern resulting from trade liberalization. The petroleum refining and coking sector emerges as the key sector contributing to the increase in GHG emissions in other East Asia and Eastern Europe. On the other hand, the scale effect is found to have a major impact on the overall increase in emissions when introducing FDI. FDI stimulates the expansion of total social output by increasing social new investment, thereby leading to widespread emission increases.

The dominance of the composition effect suggests a potential strategy for regional emission reduction within the context of B&R trade cooperation. It involves prioritizing emission reduction efforts in emission‐intensive industries where regions possess comparative advantages. In line with this approach, implementing a carbon tax emerges as a plausible and consensus response measure. Carbon tax is highly relevant, sustainable, and economically efficient, making it a suitable tool for addressing emissions in these industries.

To assess the effectiveness of a carbon tax in mitigating the GHG emissions increase resulting from trade opening, we incorporated a carbon tax into the model. Taking other East Asia as an example, which experiences significant environmental losses, we simulated the simultaneous implementation of carbon tax policies during the trade opening process. With reference to the carbon price levels observed in countries that have already implemented carbon taxes [38], two carbon price levels of US$10/ton and US$30/ton are set. Given that different carbon tax revenue recycling measures will significantly affect policy effects [39, 40], this study considers two tax recycling measures: tax revenues included in the government's general fiscal budget and used to reduce indirect taxes. The setting methods of the eight carbon tax scenarios are shown in Table 2.

**TABLE 2:** Carbon tax scenarios.

Scenario	Trade‐related settings	Carbon tax‐related settings in other East Asia (OEA)	
		Carbon price	Revenue recycling measure
BTA‐CT10‐G	Same as BTA	US$10/ton	Included in the government's general fiscal budget
MTA‐CT10‐G	Same as MTA		
BTA‐CT30‐G	Same as BTA	US$30/ton	
MTA‐CT30‐G	Same as MTA		
BTA‐CT10‐P	Same as BTA	US$10/ton	Used to reduce indirect taxes
MTA‐CT10‐P	Same as MTA		
BTA‐CT30‐P	Same as BTA	US$30/ton	
MTA‐CT30‐P	Same as MTA		

Abbreviations: BTA, bilateral trade agreement; MTA, multilateral trade agreement.

**FIGURE 5:**
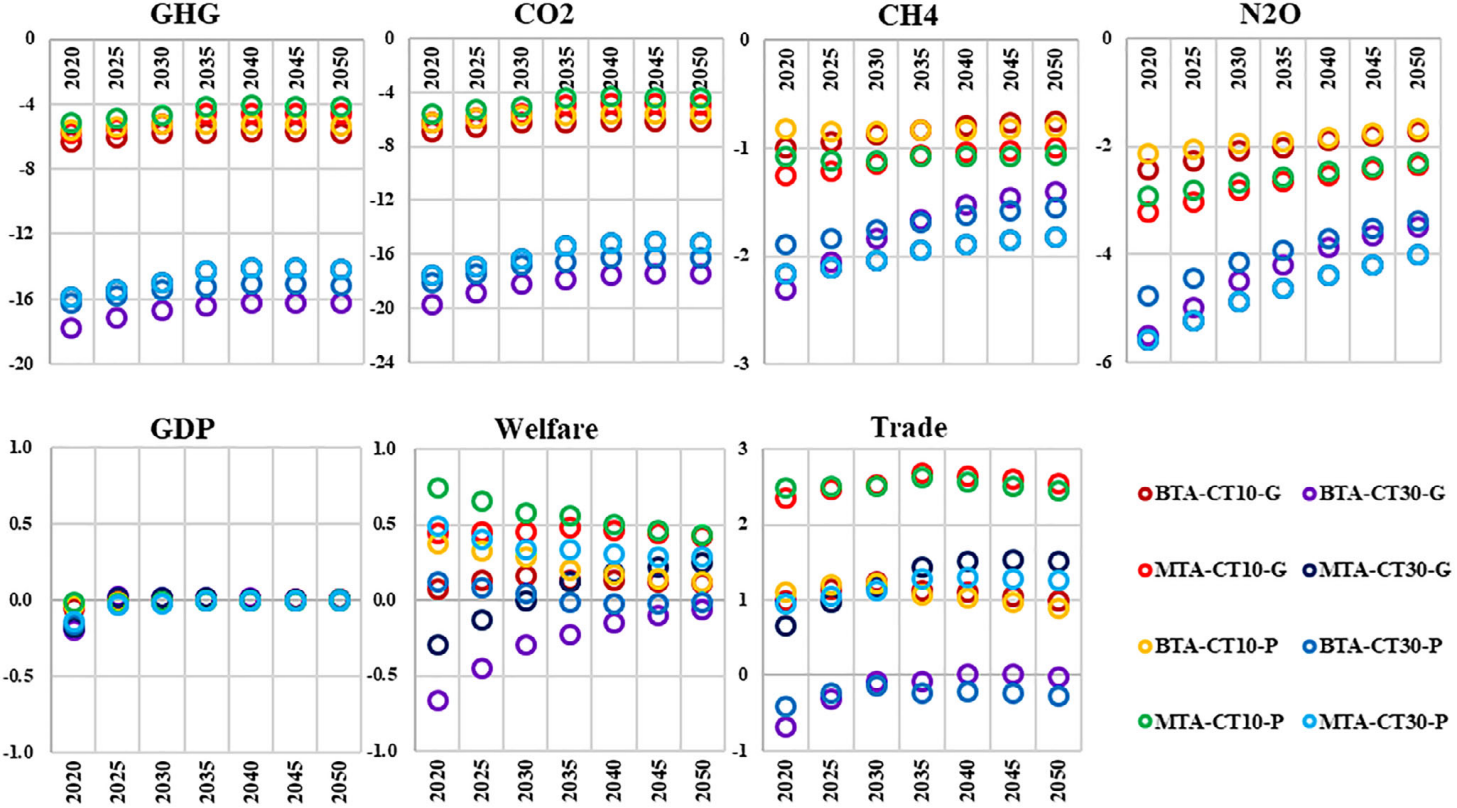
Percentage change in greenhouse gas (GHG) emissions and socio‐economic indicators of other East Asia (OEA) (%). *Note*: GHG, greenhouse gas; CO_2_, carbon dioxide; CH_4_, methane; N_2_O, nitrous oxide; GDP, gross domestic product; BTA, bilateral trade agreement; MTA, multilateral trade agreement; CT10, carbon price level of US$10/ton; CT30, carbon price level of US$30/ton; G, including carbon tax in the government's general fiscal budget; P, using carbon tax to reduce indirect taxes.

The simulation results of the carbon tax scenarios show that a desirable environmental measure in the context of opening trade in the B&R region is levying a carbon tax with a low tax rate (for example, US$10/ton simulated in this study), and its revenue is included in the government's general fiscal budget. On the one hand, this can effectively reduce the emission increase brought about by trade opening, so that all types of GHG emissions in other East Asia are below the BAU level significantly; on the other hand, the adverse impact on trade volume, GDP growth, and residents’ welfare is relatively weak (trade volume always remains at a level significantly higher than BAU; the annual GDP growth rate experiences a marginal decrease of no more than 0.2% initially, and then stabilizes at a level comparable to the trade opening scenarios; residents’ welfare surpasses that of the trade opening scenarios in the medium and long term, as depicted in Figure 5. The high carbon tax rate will significantly reduce residents’ welfare (to below the BAU level), and the revenue recycling measure of reducing indirect taxes may prolong the time of the decline of annual GDP growth in the context of the B&R trade opening. Thus, it is not suitable as a carbon tax scheme in the context of the B&R trade opening. The simulation results of the carbon tax scenarios prove the existence and effectiveness of acceptable environmental measures in the B&R trade cooperation; have reference significance for the design of the carbon tax plan; provide important insights that may be used by the relevant governments in their coordination of economic trade cooperation and environmental protection.

### 3.4 The Trade‐Off Between Social Benefits and Environmental Losses

To quantify the trade‐off between social benefits and environmental losses, and deviating from the perspective of analyzing socio‐economic conditions and environmental impacts in isolation, this study introduces a composite indicator termed Welfare Emission Intensity (WEI), as follows:

(2) $$WEI=\frac{Total\;GHG\;Emissions}{Resident\;Welfare}\;$$

**FIGURE 6:**
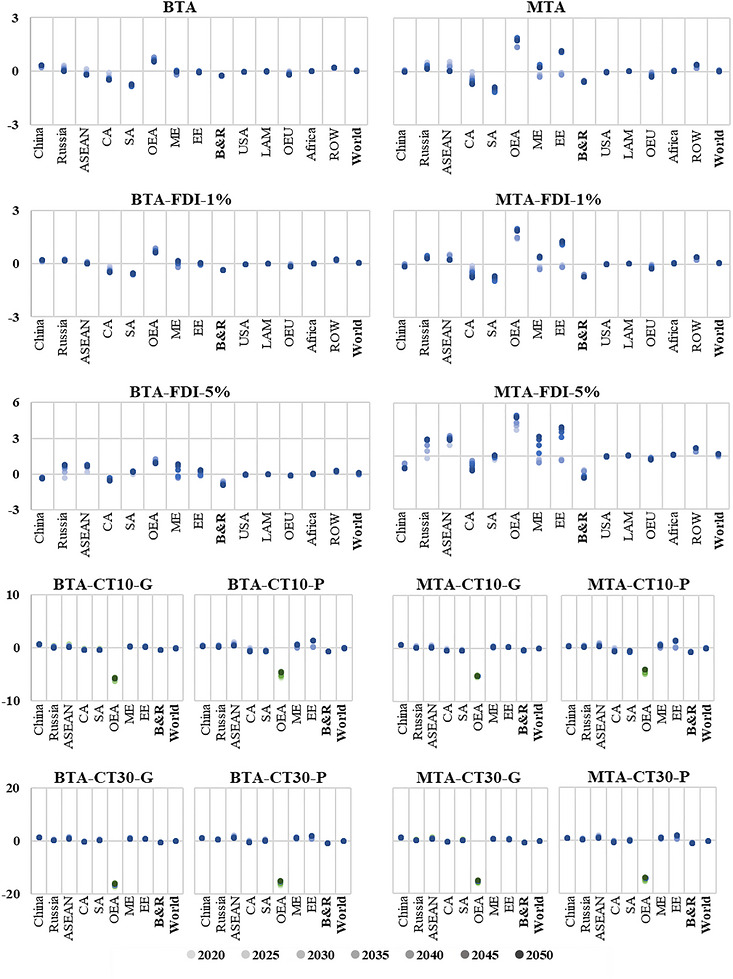
Percentage change in greenhouse gas (GHG) emissions per unit of resident welfare (%). *Note*: BTA, bilateral trade agreement; MTA, multilateral trade agreement; FDI, foreign direct investment; ASEAN, the Association of Southeast Asian Nations; CA, Central Asia; SA, South Asia; OEA, Other East Asia; ME, the Middle East; EE, Eastern Europe; USA, the United States; LAM, Latin America; OEU, other European countries; B&R, Belt and Road; ROW, the rest of the world.

The WEI is mathematically defined as the ratio of GHG emissions per unit of resident welfare. A lower WEI value indicates a lower “emission cost” for creating a unit of welfare. As shown in Figure 6, during the entire simulation period, the average WEI of the whole B&R region in the bilateral trade opening scenario drops by 0.23%–0.28% compared with BAU. In addition, the decline increases to 0.33%–0.37% after the introduction of 1% FDI (BTA‐FDI‐1% scenario), and to 0.55%‐0.93% after the introduction of 5% FDI (BTA‐FDI‐5% scenario). Compared with BAU, the WEI in the multilateral trade opening scenario decreases by 0.49%–0.57%. Moreover, the decline increases to 0.57%–0.70% after the introduction of 1% FDI (MTA‐FDI‐1% scenario), and to near or more than 1.0% after the introduction of 5% FDI (MTA‐FDI‐5% scenario). The results indicate that both trade and investment cooperation methods contribute to reducing the WEI in the B&R region. This finding helps to establish a more balanced relationship between social benefits and environmental protection.

Regarding the WEI of each specific region, other East Asia is the only region that shows a significant increase relative to BAU in the trade opening scenarios (BTA and MTA; the increase under the BTA and MTA scenarios is 0.55%–0.80%, and 1.38%–1.92%, respectively). With the welfare improvement from FDI, the WEI of other East Asia will further increase after the introduction of 1% FDI (the increase rate is 0.62%‐0.88% under the BTA‐FDI‐1% scenario, and 1.40%–1.99% under the MTA‐FDI‐1% scenario). After the introduction of 5% FDI, the WEI will increase to 0.88%–1.25% under the BTA‐FDI‐5% scenario and 1.47%–2.29% under MTA‐FDI‐5% scenario). Furthermore, the carbon tax will make this indicator drop sharply: under the desirable carbon tax scheme wherein the carbon price is US$10 and the carbon tax revenue is attributable to the government, the WEI of other East Asia is reduced by about 6%, which shows that the carbon tax can effectively reduce the “emission cost” of creating welfare.

## 4 Discussion and Conclusions

Based on a global multiregional recursive dynamic CGE model (i.e., C^3^IAM3.0/GEEPA), this study simulates and analyses trade cooperation in the B&R region, revealing the distribution of economic and environmental gains and losses in various regions of the world. Furthermore, in view of potential regional environmental losses, the introduction of a carbon tax in this study verifies the existence and effectiveness of a consensual environmental policy. Furthermore, using the composite indicator of the Welfare Emission Intensity (WEI), the coordination of socio‐economic development and environmental protection in the process of B&R economic trade cooperation is discussed. Finally, this study conducts a sensitivity analysis of key trade‐related substitution elasticities, which generally supports the robustness of the research conclusions. See Supporting Information S4 for details.

The B&R economic trade cooperation has brought socio‐economic benefits covering a wide range of participating regions, manifesting in the expansion of trade, economic growth, and an increase in residents’ welfare. The results demonstrate that trade opening promotes trade expansion and enhances residents' welfare, with multilateral trade opening having a stronger positive effect than bilateral trade opening. FDI contributes to economic growth and further improves residents' welfare. Furthermore, trade liberalization leads to a noticeable transfer in trade from non‐participating regions to participating regions and among participating regions.

Economic trade cooperation along the B&R region generates significant socio‐economic benefits while having a limited impact on global GHG emissions. However, trade liberalization can potentially result in environmental losses for specific regions, notably other East Asia and Eastern Europe. The introduction of large‐scale FDI will expand the coverage of environmental losses, and may cause other East Asia, Eastern Europe, Russia, ASEAN countries, and the Middle East to face increased GHG emissions. By decomposing the changes in GHG emissions to scale, composition, and technique effects, we found that the composition effect plays a leading role in the changes in the emission pattern brought about by trade opening (the petroleum refining and coking sector is mainly responsible for GHG emission increases in other East Asia and Eastern Europe), and the scale effect is vital in the widespread emission increase from FDI (FDI stimulates the expansion of total social output by increasing social investment).

The existence of potential environmental losses triggers discussions on the trade‐off between socio‐economic benefits and environmental protection, and at the same time prompts one to explore desirable environmental policies. By calculating the value of WEI, this study quantifies the trade‐off relationship between social benefits and environmental losses. For the vast majority of participating regions and the whole B&R region, trade opening and FDI in the B&R region are conducive to reducing the average “emission cost” of creating social benefits, and to a certain extent balance the relationship between social benefits and environmental protection. For other East Asia, the only region where the value of the indicator rises, this study verifies the existence and effectiveness of consensual environmental measures in the context of the B&R trade cooperation by introducing environmental policies that take, as an example, carbon tax into the model. It is recommended to adopt a lower carbon tax rate (for example, the US$10/ton simulated in this study) and attribute the carbon tax revenue to the government's general budget. To make these policy mixes more actionable, targeted strategies should be formulated based on regional emission drivers. For regions experiencing notable emission increases driven by the composition effect, such as other East Asia and Eastern Europe, governments are strongly advised to direct a portion of carbon tax revenues toward subsidizing green technology adoption and energy efficiency improvements in heavily affected sectors (e.g., petroleum refining and coking). Conversely, for regions where emissions rise primarily due to the FDI‐induced scale effect (e.g., Russia, ASEAN countries, and the Middle East), policymakers should implement stricter environmental screening and carbon‐intensity thresholds for incoming foreign capital. By the implementation of a reasonable and region‐specific carbon tax policy, the index value of other East Asia can be far below the BAU level. These findings prove to relevant governments that it is not necessary to make a trade‐off between economic and environmental impact in isolation. Seeking more economic cooperation and constructing a more desirable combination of policies in the process of maintaining trade openness are encouraged, to synthetically achieve SDGs on a global scale, in pursue of both socio‐economic and environmental benefits.

From a global perspective, this study analyses the temporal and spatial distribution of the environmental and economic impacts of tariff reduction and FDI. Moreover, the supporting measure (i.e., carbon tax) has been introduced. Although the methods and analysis can be used similarly to other multilateral regional cooperation, the scope of research needs both to focus on the global multiregional level and to be tailored to specific regions, which deserves a single study. Moreover, differentiated tax reduction analysis of trading countries and traded goods based on actual conditions, and trade facilitation studies that reduce the complexity of trade processes, can be further explored in the future.

## Funding

This work was supported by the National Natural Science Foundation of China (Nos. 72474021, 72293605, 72488101 and 72074022).

## Conflicts of Interest

The authors declare no conflicts of interest.

## Supporting information




**Supporting File**: gch270129‐sup‐0001‐SuppMat.zip.

## Data Availability

The data that support the findings of this study are available on request from the corresponding author. The data are not publicly available due to privacy or ethical restrictions.
