# Supplementary material for: Trade Cooperation, Environmental Protection, and Sustainability: The Belt and Road Initiative Perspective
Source: Glob Chall. 2026 Jul 16;10(7):e70129. doi: 10.1002/gch2.70129 (PMC13373936; doi:10.1002/gch2.70129)
Supplement: Supplementary file 1 — Supporting File: gch270129‐sup‐0001‐SuppMat.zip. [file GCH2-10-e70129-s001.zip › Supplementary material 4_Elasticities_sensitivity analysis.docx]

**Supplementary material 4: Elasticities and sensitivity analysis**

***1. Elasticities***

The elasticities for production and trade in this study are shown in the following Table S1.

**Table S1: Elasticities for Production and Trade**

| Sector | SigmaQ | SigmaEX | SigmaMs | SigmaEnergy | SigmaKE | SigmaX | SigmaFF |
| --- | --- | --- | --- | --- | --- | --- | --- |
| Agriculture | 4 | 2 | 5 | 0.5 | 0.9 | 0.6 | 0.6 |
| Energy | 5 | 3 | 6 | 0.5 | 0.9 | 0.6 | 0.6 |
| OtherMin | 3 | 2 | 5 | 0.5 | 0.9 | 0.6 | 0.6 |
| Elec | 0.3 | 2 | 0.3 | 0.5 | 0.9 | 0.6 | 0.6 |
| FuelGas | 0.3 | 2 | 0.3 | 0.5 | 0.9 | 0.6 | 0.6 |
| Water | 0.3 | 2 | 0.3 | 0.5 | 0.9 | 0.6 | 0.6 |
| Other | 3 | 2 | 5 | 0.5 | 0.9 | 0.6 | 0.6 |

**Note:**

(1)Elasticities’ meaning:

SigmaQ indicates the substitution elasticity between import and domestic goods;

SigmaEX indicates the transformation elasticity between export and domestic sales;

SigmaMs indicates Armington trade elasticities among imports from different regions;

SigmaEnergy indicates the substitution elasticity between energy types;

SigmaKE indiacates the substitution elasticity between capital and energy;

SigmaX indicates the substitution elasticity between labor and an aggregate of capital and energy;

SigmaFF indicates the substitution elasticity in the production process of agriculture between land and an aggregate of capital, labor, energy and intermediate inputs, and also indicates the substitution elasticity in the production process of primary energy between natural resource and an aggregate of capital, labor, energy and intermediate inputs.

(2) For sectors:

“Agriculture” includes the sectors with codes: agr;

“Energy” includes the sectors with codes: Coal, Oil, Gas, Roil;

“Other” includes the remaining sectors other than Agriculture, Energy, Elec, FuelGas and Water.

(3) Specific electricity sector’s elasticities:

SigmaCO=0.3 (Coal – Oil);

SigmaGF=0.4(Natural gas – Fuelgas);

SigmaNGR=0.4(Nuclear/Hydro resource – Value added);

SigmaVA=0.9(Labor – Capital);

SigmaFVAM=0.6(Resources of wind, solar and other – Value added & Intermediates);

SigmaRVA=0.1(Land – Resources of wind, solar and other & Value added & Intermediates);

SigmaFSVA=0.02(Fuel & Sequestration – Value added);

SigmaRO_FSVA=0.6(Resources of advanced generation technologies – Fuel & Sequestration & Value added);

SigmaStable=10(Traditional power – Nuclear/Hydro power – Advanced generation technologies power);

SigmaEWS=5(Wind, solar and other power – Traditional power & Nuclear/Hydro power & Advanced generation technologies power);

SigmaGE=0.9(Capital supply between different electricity types).

***2. Sensitivity analysis***

Considering the limitations of the CGE model when setting different elasticities, this study conducted sensitivity analysis on the key elasticities to test the robustness of the conclusions.

For the trade liberalization analysis with BRI, the substitution elasticity between import and domestic goods (SigmaQ), the transformation elasticity between export and domestic sales (SigmaEX), and the Armington trade elasticities among imports from different regions (SigmaMs) will be considered as the key elasticities. On the premise of considering the feasibility of the model, these parameters are generally set to be 50% greater or smaller than the values in the baseline scenarios, with avoiding special elasticities like 1 or 0, the adjustment of corresponding elasticities is shown in the following Table S2.

**Table S2: Elasticities for sensitivity analysis**

| Sensitivity simulations | Sector | Values | | |
| --- | --- | --- | --- | --- |
|  |  | Current values in GEEPA | Downward adjustments | Upward adjustments |
| Substitution elasticity between import and domestic goods | Agriculture | 4 | 2 | 6 |
|  | Energy | 5 | 2.5 | 7.5 |
|  | OtherMin | 3 | 1.5 | 4.5 |
|  | Elec | 0.3 | 0.3 | 0.45 |
|  | FuelGas | 0.3 | 0.3 | 0.45 |
|  | Water | 0.3 | 0.3 | 0.45 |
|  | Other | 3 | 1.5 | 4.5 |
| Transformation elasticity between export and domestic sales | Agriculture | 2 | 0.9 | 3 |
|  | Energy | 3 | 1.5 | 4.5 |
|  | OtherMin | 2 | 0.9 | 3 |
|  | Elec | 2 | 0.9 | 3 |
|  | FuelGas | 2 | 0.9 | 3 |
|  | Water | 2 | 0.9 | 3 |
|  | Other | 2 | 0.9 | 3 |
| Armington trade elasticities among imports from different regions | Agriculture | 5 | 2.5 | 7.5 |
|  | Energy | 6 | 3 | 9 |
|  | OtherMin | 5 | 2.5 | 7.5 |
|  | Elec | 0.3 | 0.3 | 0.45 |
|  | FuelGas | 0.3 | 0.3 | 0.45 |
|  | Water | 0.3 | 0.3 | 0.45 |
|  | Other | 5 | 2.5 | 7.5 |

The directions of the impacts on trade, economy and environment in each scenario remain consistent, but their extent will change slightly under each scenario with the elasticity values. For example, the greater the substitution elasticity between import and domestic goods, the greater the change in the trade volume and households’ welfare in the world, and the lower the GDP loss. In general, the results indicate that the conclusions in this study still hold under all of these tested values, except mainly that when the substitution elasticity between import and domestic goods is 50% lower.
