# Supplementary material for: Trade Cooperation, Environmental Protection, and Sustainability: The Belt and Road Initiative Perspective
Source: Glob Chall. 2026 Jul 16;10(7):e70129. doi: 10.1002/gch2.70129 (PMC13373936; doi:10.1002/gch2.70129)
Supplement: Supplementary file 1 — Supporting File: gch270129‐sup‐0001‐SuppMat.zip. [file GCH2-10-e70129-s001.zip › Supplementary material 1_Region Aggregation.docx]

**Supplementary material 1: Regional Aggregation**

| **Regions** | **Members** |
| --- | --- |
| China | China |
| Russia | Russian Federation |
| SA(South Asia) | India, Pakistan, Nepal, Bangladesh, Sri Lanka, Rest of South Asia |
| ASEAN(Association of Southeast Asian Nations) | Singapore, Malaysia, Indonesia, Thailand, Lao People's Democratic Republic, Cambodia, Viet Nam, Brunei Darussalam, Philippines, Rest of Southeast Asia |
| CA(Central Asia) | Kazakhstan, Kyrgyztan, Rest of Former Soviet Union |
| OEA(Other East Asia) | Japan, Republic of Korea, Mongolia, Rest of East Asia |
| ME(the Middle East) | Bahrain, Iran(Islamic Republic of), Israel, Jordan, Kuwait, Oman, Qatar, Saudi Arabia, Turkey, United Arab Emirates, Rest of Western Asia, Cyprus, Egypt |
| EE(Eastern Europe) | Armenia, Azerbaijan, Georgia, Czech Republic, Estonia, Greece, Hungary, Latvia, Lithuania, Poland, Slovakia, Slovenia, Albania, Bulgaria, Croatia, Romania, Belarus, Ukraine, Rest of Eastern Europe |
| USA | United States of America |
| LAM(Latin America) | Mexico, Rest of North America, Argentina, Bolivia, Brazil, Chile, Colombia, Ecuador, Paraguay, Peru, Uruguay, Venezuela (Bolivarian Republic of), Rest of South America, Costa Rica, Guatemala, Honduras, Nicaragua, Panama, El Salvador, Rest of Central America, Dominican Republic P, Jamaica, Puerto Rico, Trinidad and Tobago P, Rest of Caribbean |
| OEU(Other European onion) | Austria, Belgium, Denmark, Finland, France, Germany, Ireland, Italy, Luxembourg, Malta, Netherlands, Portugal, Spain, Sweden, United Kingdom, Rest of European Free Trade Association |
| Africa | Morocco, Tunisia, Rest of North Africa, Benin, Burkina Faso, Cameroon, Côte d'Ivoire, Ghana, Guinea, Nigeria, Senegal, Togo, Rest of Western Africa, Rest of Central Africa, South Central Africa, Ethiopia, Kenya, Madagascar, Malawi, Mauritius, Mozambique, Rwanda, United Republic of Tanzania, Uganda, Zambia, Zimbabwe, Rest of Eastern Africa, Botswana, Namibia, South Africa, Rest of South African Customs Union |
| ROW(Rest of World) | Australia, Canada, New Zealand, Switzerland, Norway, Rest of Oceania, Rest of Europe, Rest of the World |

Source: Author’s specification from GTAP Database.
