# Supplementary material for: Trade Cooperation, Environmental Protection, and Sustainability: The Belt and Road Initiative Perspective
Source: Glob Chall. 2026 Jul 16;10(7):e70129. doi: 10.1002/gch2.70129 (PMC13373936; doi:10.1002/gch2.70129)
Supplement: Supplementary file 1 — Supporting File: gch270129‐sup‐0001‐SuppMat.zip. [file GCH2-10-e70129-s001.zip › Supplementary material 2_Sector Classification.docx]

**Supplementary Material 2：Sector Classification**

| **Code** | **GEEPA Sector** | **Description** |
| --- | --- | --- |
| **1** | agr | Paddy rice, Wheat, Cereal grains nec, Vegetables, fruit, nuts, Oil seeds, Sugar cane, sugar beet, Plant-based fibers, Crops nec, Cattle,sheep, goats,horses, Animal products nec, Raw milk, Wool, silk-worm cocoons, Forestry, Fishing |
| **2** | coal | Coal |
| **3** | oil | Oil |
| **4** | gas | Gas |
| **5** | OtherMin | Minerals nec |
| **6** | fot | Meat: cattle,sheep,goats,horse, Meat products nec, Vegetable oils and fats, Dairy products, Processed rice, Sugar, Food products nec, Beverages and tobacco products |
| **7** | twl | Textiles, Wearing apparel, Leather products |
| **8** | lum | Wood products |
| **9** | ppp | Paper products, publishing |
| **10** | p_c | Petroleum, coal products |
| **11** | crp | Chemical,rubber,plastic prods |
| **12** | nmm | Mineral products nec |
| **13** | m_p | Ferrous metals, Metals nec, Metal products |
| **14** | tem | Motor vehicles and parts, Transport equipment nec, Electronic equipment, Machinery and equipment nec, Manufactures nec |
| **15** | Elec | Electricity |
| **16** | FuelGas | Gas manufacture, distribution |
| **17** | Water | Water |
| **18** | Cons | Constructions |
| **19** | TransService | Transport nec, Sea transport, Air transport |
| **20** | OthServices | Communication, Financial services nec, Insurance, Business services nec, Recreation and other services, Trade， PubAdmin/Defence/Health/Educat, Dwellings |

Source: Author’s specification from GTAP Database
