# Supplementary figures and images for: Trade Cooperation, Environmental Protection, and Sustainability: The Belt and Road Initiative Perspective
Source: Glob Chall. 2026 Jul 16;10(7):e70129. doi: 10.1002/gch2.70129 (PMC13373936; doi:10.1002/gch2.70129)

**Supplementary material 3: The contributions of Scale, composition and technique effects (%)**


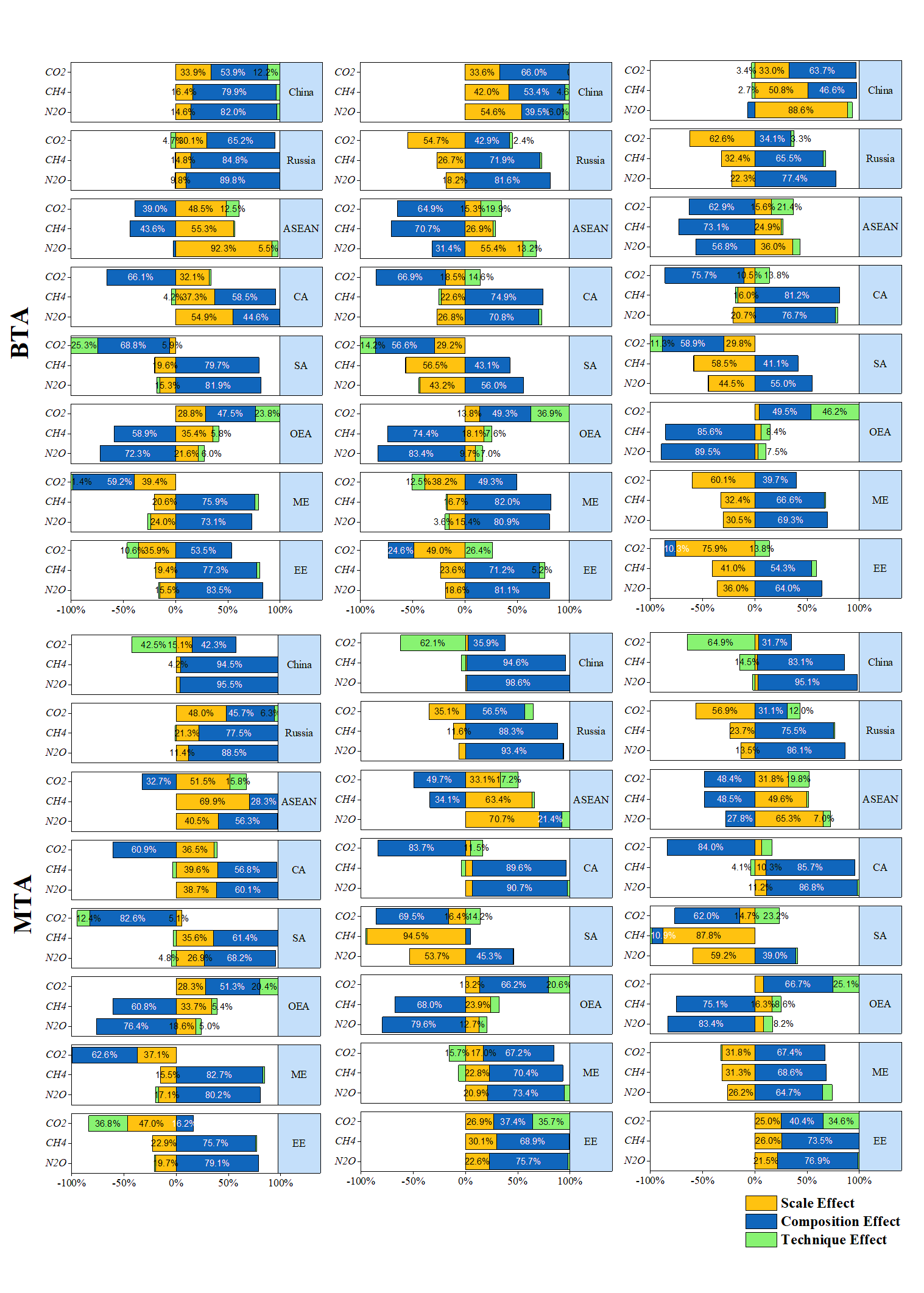


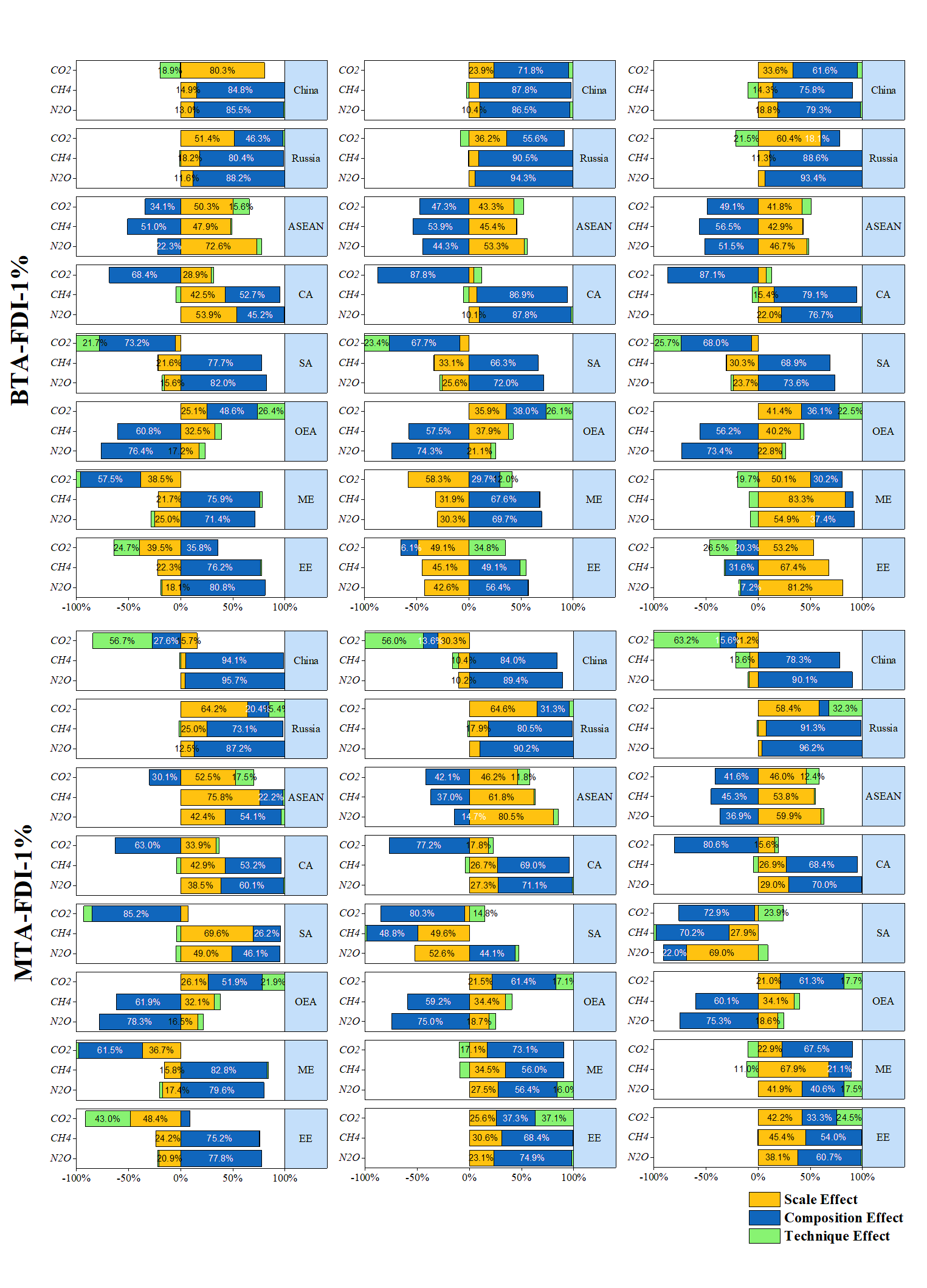


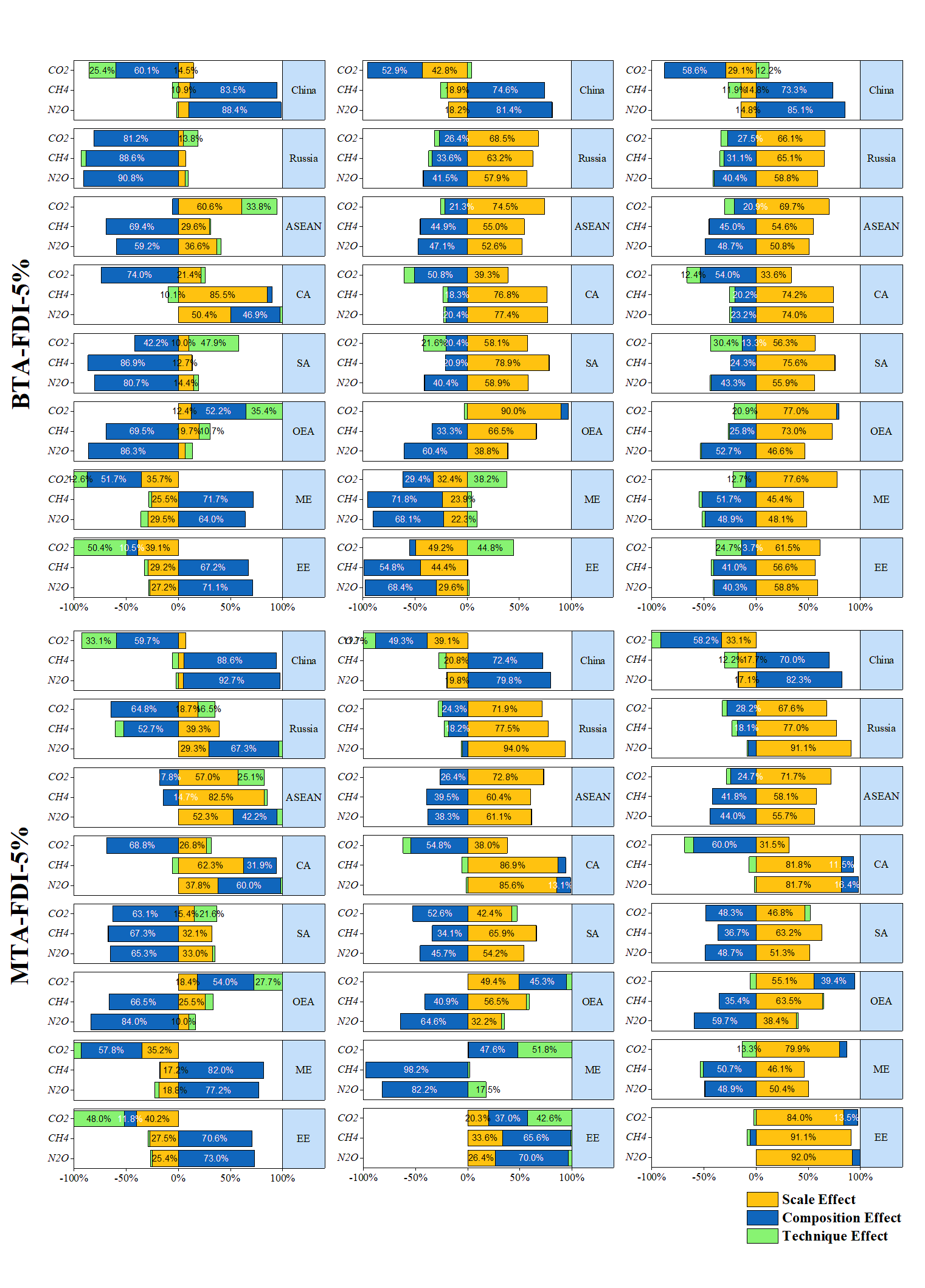

Supplement: Supplementary file 1 — Supporting File: gch270129‐sup‐0001‐SuppMat.zip. [file GCH2-10-e70129-s001.zip › Supplementary material 3_Decomposition results.docx]
